# Antifungal properties of (2S, 4R)-Ketoconazole sulfonamide analogs

**DOI:** 10.3389/fddsv.2022.1000827

**Published:** 2022-08-29

**Authors:** Benjamin E. Blass, Sumant Puri, Rishabh Sharma, Brian M. Day

**Affiliations:** 1 Department of Pharmaceutical Sciences, Temple University School of Pharmacy, Philadelphia, PA, United States,; 2 Oral Microbiome Research Laboratory, Kornberg School of Dentistry, Temple University, Philadelphia, PA, United Stated

**Keywords:** antifungal agent, antimicrobial agents, ketoconazole, *Candida albicans*, *Candida glabrata*

## Abstract

Invasive candidiasis remains a significant health concern, as it is associated with a high mortality risk. In addition, the risk of infection is significantly elevated in immunocompromised patients such as those with HIV, cancer, or those taking imcmunosuppressive drugs as a result of organ transplantation. The majority of these cases are caused by *C. albicans*, and *C. glabrata* is the second most common cause. These infections are typically treated using approved antifungal agents, but the rise of drug-resistant fungi is a serious concern. As part of our on-going effort to identify novel antifungal agents, we have studied the *in vitro* antifungal properties of a series of sulfonamide analogs of (2S, 4R)-Ketoconazole. Herein we report on the *in vitro* activity against the key fungal pathogens *C. albicans*, and *C. glabrata*.

## Introduction

According to the U.S. Center for Disease Control (CDC), invasive candidiasis remains a significant health issue. The average incidence rate of candidemia in 2013–2017 was 9 per 100,000, and it is estimated that ~25,000 new cases occur annually nationwide (Tsay et al., 2018). The annual global incidence of invasive candidiasis is ~750,000 (Bongomin et al., 2017). Candida is one of the most common causes of bloodstream infections in the U.S (Magill et al., 2018). In addition, candidemia is associated with a high mortality risk. The U.S. CDC reported that between 2012 and 2016, ~25% of candidemia patients died while hospitalized (US CDC website 2022b). Though less common, invasive candidiasis can also occur in various organs in the absence of bloodstream infection. The danger of fungal infection is significantly elevated when the immune system is compromised by viral infection (e.g. Human Immunodeficiency Virus (HIV)/Acquired Immunodeficiency Syndrome (AIDS)), immunosuppressive agents designed to support organ transplants, anticancer agents, and antibody therapies used to treat autoimmune disorders. Risks are also elevated when natural barriers are disrupted (e.g., surgery, burns) (Monk et al., 2020).

Importantly, nearly 95% of all forms of invasive *candida* infection in the U.S. are caused by 5 *candida* species (*C. albicans*, *C. glabrata*, *C. parapsilosis*, *C. tropicalis*, and *C. krusei*.). *C. albicans* is the leading cause of *candida* infection in the U.S., but non-*C albicans* infections account for nearly 2/3 of candidemia case in the U.S. (US CDC website (2022b). *C. glabrata* is the second most common invasive fungal species, accounting for 15–25% of clinical isolates in the U.S. (Pfaller and Diekema, 2007; McCarty and Pappas 2016). This species is associated with high morbidity and mortality (40–60%) (Timmermans et al., 2018). In addition, there has also been a steady increase in fungal drug resistance (Roemer and Krysan, 2014). Much like antibacterial agents, prolonged and/or repeated clinical use of antifungal agents induces resistance pathways and the emergence of drug-resistant fungi (Sanglard et al., 1999; Vermitsky and Edlind, 2004; Chau et al., 2006). The broad use of antifungal agents in the agrochemical sector has also been linked to the development of cross-resistance to antifungal agents used in medicine (Snelders et al., 2008; Snelders et al. 2011; Snelders et al. 2015). Drug resistance is increasingly problematic with *C. albicans* as well as non-albicans Candida species such as *C. glabrata* (Vallabhaneni et al., 2015; Kolaczkowska and Kolaczkowski. 2016).

**FIGURE 1:**
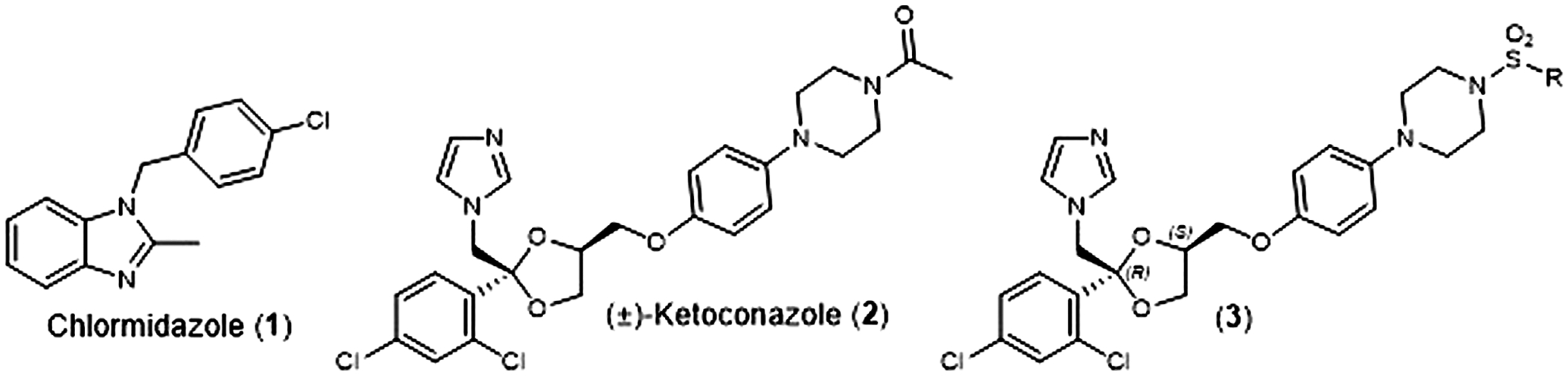
Structures of Chlormidazole (**1**), (±)-Ketoconazole (**2**), and (2S, 4R)-Ketoconazole sulfonamide analogs (**3**).

Fungal infections are typically treated with medications from one of four class of antifungal agents; polyenes, echinocandins, polyenes, and azoles (Griffith et al. (2020)). In 1958. Chlormidazole (**1**) (Figure 1) became the first clinically available azole antifungal agent, and in 1981, Janssen Pharmaceuticals received U.S. Food and Drug Administration (FDA) approval for the first orally available antifungal agent, (±)-Ketoconazole (**2**) (Figure 1). For nearly a decade, this medication was the only orally available antifungal agent (Maertens, 2004), but it has been conclusively linked to potentially severe safety issues (hepatoxicity, adrenal insufficiency, and Cytochrome P450 3A4 (Cyp3A4) mediated drug/drug interaction). This led the FDA to severely restricted oral use of (±)-Ketoconazole (**2**) in 2013 (FDA Website, 2022).

As part of our effort to address the need for new antifungal agents, we have been studying the chemical space surround (±)-Ketoconazole (**2**). We were surprised to discover that since its initial discovery (Backx et al., 1979), there have been very few reports of direct analogs of (±)-Ketoconazole (**2**) or its individual enantiomers. In 1992, D. M. Rotstein et al. reported on the ability of the 4 stereoisomers for (±)-Ketoconazole (**2**) to inhibit lanosterol 14α-demthylase (Cytochrome P450 51a, Cyp51a), the biochemical target that gives rise to the azole’s antifungal properties. They found that (2S, 4R)-Ketoconazole was the most potent inhibitor of the four (Rotstein et al., 1992). Separately, we have reported on the synthesis and evaluation of a series of (2S, 4R)-Ketoconazole sulfonamide analogs (**3**) (Figure 1) as potential treatments for metabolic syndrome (Blass et al., 2016). Herein, we report the *in vitro* antifungal properties of these compounds against *C. albicans* and *C. glabrata*.

## Material and methods

**FIGURE 2:**
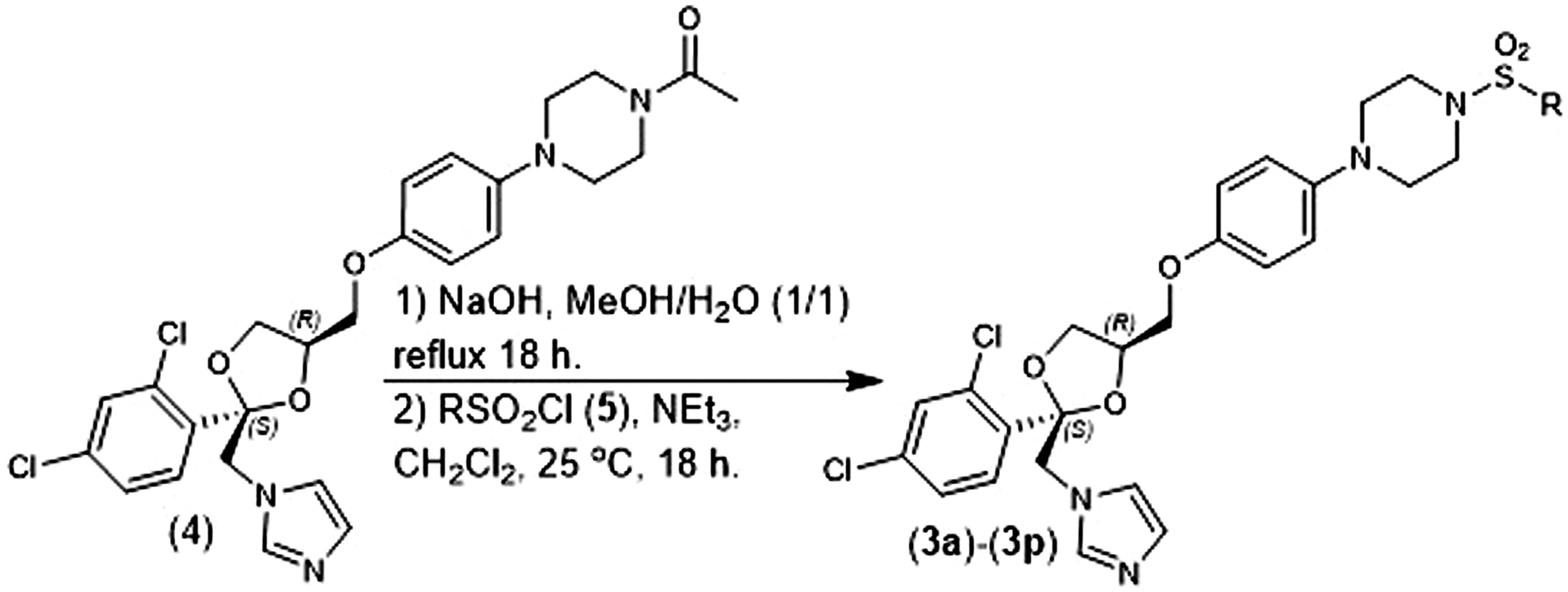
Synthesis of (2S, 4R)-Ketoconazole sulfonamide analogs.

Chemical compounds: The (2S, 4R)-Ketoconazole sulfonamide analogs were prepared according to our previously reported methods (Blass et al., 2016). Briefly, (2S, 4R)-Ketoconazole (**4**) was hydrolyzed with NaOH in refluxing methanol/water and the resulting product was converted to a sulfonamide (**3a-3l**) using the corresponding sulfonyl chloride (**5**) in the presence of NEt_3_ and CH_2_Cl_2_ (Figure 2). Amphotercin B was purchased from ACTGene Inc. (Catalog number R1397–1 g). (±)-ketoconazole we purchased from Combi-Blocks Inc. (Catalog number QA-7778–1 g).

Broth microdilution susceptibility test: The minimum inhibitory concentrations (MIC_75_) were determined by the broth microdilution method according to the Clinical and Laboratory Standards Institute (CLSI) M27-A4 standard (CLSI, 2017)). Briefly, a suspension of *C. albicans* or *C. glabrata* culture (~0.5–2.5 × 10^3^ cells/ml) was resuspended in RPMI 1640 media (Fisher Catalog number 11875093). The resuspended culture was further incubated with 100 μl of two-fold serially diluted test compounds (100–0.8 μM and 1.0 μM to 7 nM) at 30 °C for 24–48 h. Positive and negative control (wells without antifungal agents and wells without yeast) were conducted. Increased optical density (OD) corresponds to the Candida growth and was quantified by comparison with untreated Candida control samples. The MIC_75_ of test compounds was determined visually and spectrophotometrically (OD600) after 24–48 h. Experiments were run in triplicate, and data was reported as a mean.

Cyp3A4 inhibition assay: Human Cyp3A4 Inhibition assay: An IC_50_ for inhibition human Cyp3A4 metabolism of midazolam to 1-OH-midazolam was determined with 10 concentrations of test compounds (half-log serial dilutions; duplicate points). Assays were conducted in 1 ml 96 well polypropylene plates containing 100 μL of 100 mM potassium phosphate (pH 7.4), 3 mM MgCl_2_, 2 μM midazolam, 1 mM NADPH (tetra Na salt), Corning Gentest supersomes (product 456202: 3 pmol/ml human Cyp3A4, 47 nmol/min human P450 reductase activity, 14 pmol human cytochrome b5; and product 456201: insect control microsome protein; total final microsome protein concentration 0.15 mg/ml) and test compound. All components except NADPH were added to a prewarmed plate and reactions were initiated by adding NADPH. After 30 min at 37°C, reactions were terminated with 200 μL acetonitrile containing 30 μM prednisone. After centrifugation for 10 min at 2200 × g, 165 μL of supernatants were transferred to analysis plate. Samples were analyzed for 1-OH-midazalam concentration, using prednisone as the internal standard, using a Waters Aquity UPLC in tandem with a Xevo TQ MS mass spectrometer (BEH C18 column; 5–95% acetonitrile gradient with 0.1% formic acid; ESP + mode). IC_50_’s were determined using GraphPad’s Prism v 5.04 nonlinear curve fitting program.

## Results

The *in vitro* antifungal activity of our of (2S, 4R)-Ketoconazole sulfonamide analogs (**3a**) –(**3l**) against *C. albicans* and *C. glabrata* is described in Table 1. Replacing the acetamide of (2S, 4R)-Ketoconazole (**4**) with small, linear suflonamides (**3a**-**3c**) produced compounds whose *in vitro* potency against the test fungi was maintained (*C. albicans* MIC_75_ = 125 nM, *C. glabrata* MIC_75_ = 500 nM). Insertion of a branch point as seen in the isopropyl analog (**3d**), however, led to a complete loss of *in vitro* antifungal activity. Constraining the branch point through the formation of the corresponding cyclopropane ring (**3e**) restore *in vitro* antifungal activity against both species (*C. albicans* MIC_75_ = 800 nM, *C. glabrata* MIC_75_ = 800 nM), but not to the levels observed in (**3a**) –(**3c**). Insertion of an additional methylene unit between the cyclopropane ring and the sulfonamide moiety (**3f**) produced a significant loss *in vitro* activity against *C. glabrata* (MIC_75_ = 25,000 nM), but activity against *C. albicans* (MIC_75_ = 800 nM) was unchanged in comparison to (**3d**).

The addition of polar groups to the sulfonamide moiety produced mixed results. Appending a cyano group on the sulfonamide moiety (**3g**) provided a compound with *in vitro* antifungal activity comparable to that of (±)-Ketoconazole (**2**) (*C. albicans* MIC_75_ = 250 nM, *C. glabrata* MIC_75_ = 500 nM), but adding a methyl sulphone unit (**3h**) caused *in vitro* antifungal activity to drop substantially (*C. albicans* MIC_75_ = 800 nM, *C. glabrata* MIC_75_ = 6250 nM).

Fluorination of the small, linear sulfonamides produced some surprising results. The CF_3_ analog (**3i**) was less potent (*C. albicans* MIC_75_ = 800 nM, *C. glabrata* MIC_75_ = 1600 nM) than the parent of series (**3a**). Similar results were observed when the ethyl sulfonamide and n-propyl sulfonamide where capped with a CF_3_ moiety (**3j** *C. albicans* MIC_75_ = 1600 nM, *C. glabrata* MIC_75_ = 800 nM, **3k** *C. albicans* MIC_75_ = 1600 nM, *C. glabrata* no effect). Interestingly, capping the ethyl sulfonamide with a difluoro unit (**3l**), however, produced a significant increase *in vitro* antifungal activity against both species examined (*C. albicans* MIC_75_ = 62 nM, *C. glabrata* MIC_75_ = 250 nM). Importantly, the *in vitro* antifungal activity of (**3l**) exceeded that of both Amphotericin B and (±)-Ketoconazole against *C. albicans* (MIC_75_ = 250 nM) and matched their activity against *C. glabrata* (MIC_75_ = 250 nM).

We also determined the capacity of our compounds to inhibit human Cyp3A4. As noted above, the use of (±)-Ketoconazole (**2**) was heavily restricted in 2013 based in part on its ability to cause drug/drug interaction *via* inhibition of this key metabolic enzyme (FDA Website, 2022). In our hands, (±)-Ketoconazole (**2**) and (2S, 4R)-Ketoconazole (**4**) displayed potent Cyp3A4 inhibition (IC_50_ = 133 and 146 nM respectively). Our (2S, 4R)-Ketoconazole sulfonamide analogs (**3a**-**3l**) displayed moderate to potent Cyp3A4 inhibition (IC_50_ = 75.6–450 nM). The n-propyl analog (**3c**) was the most potent (IC_50_ = 75.6 nM), while the trifluoroethyl analog (**3j**) was the least potent of the set (IC_50_ = 450 nM).

## Discussion

As noted above, *C. albicans* and *C. glabrata* are responsible for a large portion of Candida infection in the US and represent a serious health threat (US CDC website, 2022a). Blood stream infection of either of these organisms are associated with high mortality (Timmermans et al., 2018). Immunocompromised patients are at an even greater risk should they be unfortunate enough to experience infections with *C. albicans, C. glabrata*, or other *candida* species (Monk et al., 2020). In addition, the rise of drug resistant fungi (Sanglard et al., 1999; Vermitsky and Edlind, 2004; Chau et al., 2006) suggests that the current staple of antifungal agents may eventually be insufficient to meet patient needs. Similar to antibiotics and antimicrobial agents, the appearance of clinically relevant drug resistant fungi has been driven by prolonged use of antifungal agent in clinical setting as well as in the agricultural sector (Snelders et al., 2008; Snelders et al. 2011; Snelders et al. 2015).

Our studies focused on the further exploration of the chemical space surrounding (±)-Ketoconazole (**2**) began with the observation that the initial disclosure of this antifungal agent (Beckx 1979) described only a handful of analogs. This initial disclosure predates the advent of both high through put chemistry and high throughput screening, which may explain the limited nature of the original disclosure. Surprisingly, we found that the literature contained very few additional disclosures of (±)-Ketoconazole analogs, and we viewed this as an opportunity to explore the chemical space surrounding this antifungal agent. Specifically, we have demonstrated that the acetamide of (2S, 4R)-Ketoconazole (**4**) can be replaced with sulfonamides (**3a**-**3l**) to produce compounds that have *in vitro* antifungal properties with respect to *C. albicans* and *C. glabrata*. We have also found that similar to (±)-Ketoconazole (**2**), these compounds inhibit human Cyp3A4 (IC_50_ = 75.6–450 nM), a key metabolic enzyme that has been linked to drug/drug interactions (Molenaar-Kuijsten et al., 2021). Interestingly, we noted a divergence between antifungal activity and Cyp3A4 inhibition, Specifically, we observed that our most potent *in vitro* antifungal agent (**3l**) demonstrated a wider window between Cyp3A4 inhibition and its activity against *C. albicans* than (±)-Ketoconazole (**2**) (2.6-fold versus 0.5-fold). This 5-fold improvement in selectivity suggests that it may be possible to identify analogs with a wide enough separation to avoid the risk of Cyp3A4 mediated drug/drug interaction. Additional studies will be required to determine the true potential of this opportunity.

## Figures and Tables

**TABLE 1:** *In vitro* antifungal activity of (3a)—(3l) against *C. albicans* and *C. glabrata*.

Entry	R	MIC_75_ (nM)		CYP 3A4
		*C. albicans*	*C. glabrata*	IC_50_ (nM)
Amphotericin B	N/A	250	250	ND
2	N/A	250	500	133
4	N/A	125	500	146
3a	SO_2_Me	125	500	158
3b	SO_2_Et	125	500	162
3c	SO_2_-n-Prop	125	500	75.6
3d	SO_2_-i-Prop	N.E.	N.E.	198
3e	SO_2_-cyc-Prop	800	800	185
3f	SO_2_CH_2_-iProp	800	25000	246
3g	SO_2_CH_2_CN	250	500	153
3h	SO_2_CH_2_SO_2_CH_3_	800	6250	151
3i	SO_2_CF_3_	800	1600	336
3j	SO_2_CH_2_CF_3_	1600	800	450
3k	SO_2_CH_2_CH_2_CF_3_	1600	N.E.	249
3l	SO_2_CH_2_CHF_2_	62	250	162

* N.E., no effect, N/A = not applicable, ND, not determined.

## Data Availability

The original contributions presented in the study are included in the article/Supplementary material, further inquiries can be directed to the corresponding authors.
